# Design and psychometric evaluation of sociocultural scale predicting the incidence of road traffic crashes in drivers

**DOI:** 10.5249/jivr.v14i3.1707

**Published:** 2022-07

**Authors:** Zahra Haghdoust, Gholamreza Masoumi, Shandiz Moslehi, Abbas Ebadi, Davoud Khorasani Zavareh

**Affiliations:** ^*a*^ Department of Health in Disasters and Emergencies, School of Health Management and Information Sciences, Iran University of Medical Sciences, Tehran, Iran.; ^*b*^ Health in Disasters and Emergencies Research Center, University of Social Welfare and Rehabilitation, Tehran, Iran.; ^*c*^ Department of Emergency Medicine, Trauma and Injury Research Center, Iran University of Medical Sciences, Tehran, Iran.; ^*d*^ Health Management and Economics Research Center, Health Management Research Institute, Iran University of Medical Sciences, Tehran, Iran.; ^*e*^ Behavioral Sciences Research Center, Life Style Institute, Baqiyatallah University of Medical Sciences, Tehran, Iran. Nursing Faculty, Baqiyatallah University of Medical Sciences, Tehran, Iran.; ^*f*^ Workplace Health Promotion Research Center, Department of Health in Disasters and Emergencies, School of Public Health and Safety, Shahid Beheshti University of Medical Sciences, Tehran, Iran.

**Keywords:** Sociocultural Factors, Predictors, Road Traffic Crash, Validity, Reliability

## Abstract

**Background:**

Various factors are involved in the occurrence of Road Traffic Crashes (RTCs), one of the most important of these are human factors that can be greatly influenced by the specific sociocultural bases of the drivers. So far, there has not been a scale for measuring Sociocultural Factors (SCFs) predicting the occurrence of RTCs in Iranian drivers. Therefore, the present study was conducted to design and to do psychometric evaluation of a scale for measuring SCFs predicting the occurrence of RTCs in drivers.

**Methods:**

This exploratory sequential mixed method was carried out in three phases. In phases 1 and 2, an initial items pool was created based on systematic literature review (phase 1), and semi structured interviews (phase 2). In phase 3, the initial scales were validated using face and content validities. Then, principal component analysis and confirmatory factor analysis were performed to assess the construct validity. Finally, the reliability of the scale was evaluated by examining internal consistency and stability.

**Results:**

The scale content validity index was 0.92. Principal component analysis showed seven factors with 27 items, which explain 55.56% of the total variance. In confirmatory factor analysis, model fit indices were satisfactory. Discriminant analysis was also able to distinguish between two groups of accident-involved drivers and accident-free drivers (P less than 0.0001). The reliability of the scale by Cronbach's alpha, Theta, Omega and intra-class correlation coefficients was 0.82, 0.96, 3.07, and 0.80, respectively.

**Conclusions:**

This scale can be used as a valid and reliable scale to evaluate the SCFs predicting the occur-rence of RTCs in drivers. Furthermore, the findings of this study will be useful in identifying and planning to reduce RTCs, especially in accident-prone drivers.

## Introduction

Various studies have indicated that human, vehicle, road conditions, and environmental factors are involved in causing Road Traffic Crashes (RTCs). Based on experiences of Low- and Middle-Income Countries (LMICs), the contribution of human factors including Sociocultural Factors (SCFs) in RTCs varies up to 95%.^1,2^ The researchers suggested that RTCs can be predicted and prevented in many cases, by identifying and changing SCFs.^3,4^ The most practical solution to identify and measure SCFs is to use instruments that can provide a better understanding the behavior of at-risk population. These instruments can play an important role in preventing RTCs and improving the community health by influencing social determinants of health, which are headed by RTCs. Furthermore, they can assist health and emergency managers and policymakers in identifying high-risk drivers in the risk analysis step. 

RTCs are the leading cause of injury and the second cause of death in Iran.^5^ The deaths and damages caused by RTCs in Iran are as high as in other LMICs.^6^ Despite the efforts of researchers and policymakers to identify the human factors predicting RTCs, investment in road safety development has not commensurate with the problem scale yet.^7^ First of all, most of the available instruments for predicting RTCs are designed to measure various aspects such as attitude,^8^ personality,^9^ behavior^10^ and performance^11^ of drivers; and less attention has been paid to SCFs. Second, according to the past research, these instruments are simple and declining dramatically. Since those measurement scales don't take into account many of the issues of the new age, such as fatigue and cell phone use, which can negatively affect driver performance,^12^ therefore, there is a lack of studies to design and develop indigenous instrument for measuring SCFs predicting RTCs in Iranian drivers. Therefore, it remains inconclusive to identify this growing public health threat on a national scale due to lack of reliable and systematic data. Depending on indigenous conditions of each country, there are various models and instruments to identify human factors contributing to RTCs in drivers.^8,10^ However, an individual country’s programs cannot meet the needs of other countries, because there are some differences in the structure of instruments due to the diverse sociocultural context of countries. Nordfjærn et al., in the same way, pointed out that the indigenous conditions of each community have different effects on the incidence of RTCs due to having different sociocultural structure and driving infrastructure.^13^

**Aim of the present study**

The purpose of the present study was to provide a reliable and valid instrument for assessing SCFs contributing to RTCs among Iranian drivers. In order to achieve this goal, three phases were conducted. The first and second phases were designed in the forms of systematic literature review and qualitative study, respectively, to clarify and explain the SCF predicting RTCs, identify the dimensions related to them, and produce items. In the quantitative phase (Phase 3), the items of the initial instrument entered the psychometric stages to evaluate the validity and reliability.

## Materials and Methods

An exploratory sequential mixed methods was used to design the scale from December 2019 to June 2021.

***Study design***

This study was designed in three phases. The first phase was a systematic literature review, the second phase was a qualitative study, and the third phase was a quantitative study and psychometrics of scale. The last phase included the stages of face, content and construct validity, internal consistency reliability and intraclass correlation coefficient. (Appendix 1)

**Appendix 1:**
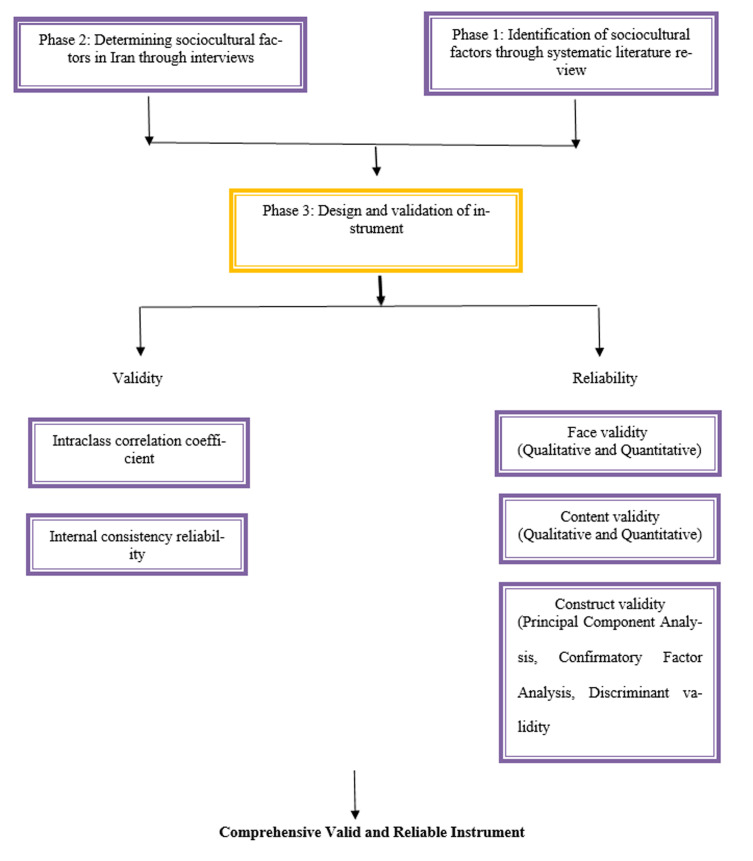
Steps of Research

***Item generation***

In order to generate items for the scale, a systematic literature review and a qualitative study were conducted to form the concept of SCFs in drivers. These are as follows:

**1- Systematic literature review (phase 1): **A purposeful electronic search was conducted through international data bases including PubMed, Web of Science, Scopus, ProQuest, Cochran Library for English publications; and Google Scholar, and national data bases such as Magiran, Irandoc, Scientific Information Database (SID), Islamic World Science Citation Center (ISC), Noormags for Persian publications, in order to develop the concept of SCFs and to generate questionnaire items. Key journals based on Scopus search, references list of entered articles, gray literature and website of related organizations were hand-searched to find more appropriate studies and to ensure comprehensiveness of the search. The results of this phase of the study were in line with the publication of Iran Medical Journal of the Islamic Republic of Iran.^14^

**2- Qualitative study (phase 2): **Face-to-face semi-structured interviews were conducted to clarify SCFs influencing RTCs based on the perception of experts and car drivers. The study’s participants included 25 specialists in road traffic, psychology and sociology with experience about effective factors in the occurrence of RTCs, prevention and treatment of injuries, and safety promotion (male=72%; female=28%), and 50 car drivers with a 10-year driving experience and 3 or more types of accidents leading to human injury, vehicle damages, and death), who were selected using purposive sampling with maximum variety (age, marital status, education, number of children, place of residence, economic status, type of vehicle, vehicle ownership, smoking and drinking consumption). Each interview lasted for 20-54 minutes, with an average duration of 34 minutes. The interviews were immediately transcribed verbatim. Codes were extracted after reading the transcribed text several times using the Graneheim and Lundman approach.^15^ In this method, the texts were converted into meaningful units, condensed meaning units and codes. Then, the codes were classified according to their similarities and differences in the subcategories. This process continued for all interviews until formation of the main categories. To identify ideas, the data collection and analysis process began simultaneously. The participants expressed their consent in a written form for taking part in the interview and audio recording. In addition, they were assured of the confidentiality of their private information.

**3- Item pool: **A total of 110 items were generated from the first and second phases. Of these, 39 items from systematic literature review, 41 items from interview with specialists, and 30 items from interview with drivers were extracted.

***Item reduction***

At this stage, the items were revised during joint meetings with the research team, and overlapping or highly similar items were merged or deleted. Thus, the items were reduced to 89. Then, the initial version of the scale with 89 items entered the psychometric stages.

***Quantitative phase (phase 3)***

In the quantitative phase, the validity and reliability of the scale were determined, and the psychometric properties of the scale were evaluated.

***Face and content validity***

Face validity was determined using qualitative and quantitative methods. In the qualitative step, 30 car drivers with a valid driver's license and a history of three accidents (accidents leading to human injury, vehicle damages, or death) in the last three years, were selected through purposive sampling by referring to parking, passenger stations, terminals and travel agencies in Tehran (Capital of Iran) and Guilan (North of Iran) provinces. They evaluated the scale items in terms of difficulty, relevance and ambiguity. In the quantitative step, the impact score indicator (frequency × suitable) was used. Thus, another 30 drivers assessed the suitability of each item based on 5-point Likert scale (from "not suitable at all "to "it's perfectly suitable"). The items with an impact score more then 1.5 were considered appropriate.^16^

Then, content validity of the instrument was examined by expert panel, which included 10 specialists in psychology, sociology, road traffic, as well as disasters and emergencies. In the qualitative step, they evaluated the questionnaire in terms of grammar, use of appropriate words, item allocation and scoring. In the quantitative step, 10 other specialists with the same characteristics calculated Content Validity Ratio (CVR) based on Lawshe table (CVR> 0.62), ^17^ Content Validity Index (CVI), Item Content Validity Index (ICVI> 0.78), Scale Content Validity Index/Average (SCVI/Ave>0.8) and Kappa value (Kappa> 0.74). Finally, a decision was made to remove and modify the items based on the above-mentioned indicators and expert comments. 

***Item analysis***

In the item analysis, a preliminary study was conducted on 30 car drivers before performing factor analysis to evaluate the reliability, the corrected item-total correlation, inter-item correlation and scale, if item deleted.

***Construct validity***

To evaluate the construct validity, the following procedures were performed: 

***1- Principal Component Analysis: *** Principal Component Analysis (PCA) is one of the best methods used to evaluate the construct validity. As a general rule, a sample size of 300 cases was considered suitable. Therefore, 300 car drivers who met the inclusion criteria (driving as a job, driving with a valid license, and expressing the consent for participate in the study) were selected by convenient sampling. The samples included 271 male (90.3%) and 29 female drivers (9.7%). The participants’ ages ranged from 23 to 76 years (M=46.21, SD=10.747). The number of years of driving experience since participants obtained their driver's licenses ranged from 4 to 56 years (M=22.04, SD=10.363). In terms of education level, 0.3% of participants were illiterate, 6% had an elementary education, 20% had a secondary and high education, 47.7% had a high school diploma, and 26% had a university education. 

Components were extracted by Principal Component Analysis (PCA) with eigenvalues >1 and the Varimax rotation method. Factor loading values <0.4 were removed from the output. Sampling adequacy was tested using Kaiser-Meyer-Olkin (KMO), the recommended value was at least 0.6, and the Bartlett test was suggested for showing significance.^18^

After extracting the components in the PCA, other 200 drivers with similar features to PCA were evaluated using Confirmatory Factor Analysis (CFA) and goodness of fit indices, to confirm the construct validity. The sample included 163 male (81.5%) and 37 female drivers (18.5%). The participants’ ages ranged from 28 to 70 years (M=42.68, SD=9.815). Years of driving experience since participants acquired their driver's licenses ranged from 10 to 50 years (M=18.40, SD=9.051). In terms of education level, 0.5% of participants were illiterate, 5.5% had an elementary education, 22% had a secondary and high education, 50% had a high school diploma, and 22% had a university education. Twelve fitness indices were used to assess the model fit of the SCFs, including the goodness-of-fit index (GFI), Adjusted Goodness of Fit Index (AGFI), Standardized Root Mean Square Residual (SRMR), Relative Fit Index (RFI), Incremental Fit Index (IFI), Comparative Fit Index (CFI), Parsimony Normed Fit Index (PNFI), Non-Normed Fit Index (NNFI), Normed Fit Index (NFI), Minimum Discrepancy Function by Degrees of Freedom divided (CMIN/DF), Root Mean Square Error of Approximation (RMSEA) and Chi-squared P-value ( χ 2 P-value). The cut-off values were set at >0.90 for the NFI, NNFI, CFI, IFI, RFI and GFI; >0.8 for the AGFI; >0.05 for the χ 2 P-value; <3 for CMIN/DF; >0.5 for the PNFI; <0.1 for the SRMR; and <0.08 for the RMSEA.^19^

***2- Discriminant Validity: ***For discriminant validity, the finalized scale after factor analysis was given to two groups of 30 drivers selected by purposive sampling; and the results were compared with independent t-test.

The first group consisted of at fault accident-involved drivers with more than 10 years of driving experience and a history of three accidents (accidents leading to human injury, vehicle damages, and death) in the last three years. The second group included accident-free drivers having more than 10 years of driving experience and car insurance discounts. The first group’s drivers were selected by referring to the parking, passenger stations, terminals and travel agencies of Tehran and Guilan provinces; and the second group’s drivers were selected by referring to the insurance offices. The drivers who had experienced serious vehicle damages in accidents but paid the damage themselves due to not losing the car insurance discount were excluded from the study with self-reporting. Finally, the responses of the two groups were compared. 

***Reliability***

An alpha value ≥0.7 was considered to measure internal consistency reliability.^20^ Theta, and Omega coefficients were calculated. To assess the relative reliability by intraclass correlation coefficient (ICC), 30 drivers completed the questionnaires in two steps with an interval of two weeks. Correlation coefficient ≥0.8 was considered satisfactory.^21^

***Absolute reliability***

The Standard Error of Measurement (SEM) and Minimal Detectable Change (MDC) were calculated to measure absolute reliability. The following equation was used to measure the standard error of measurement: SEM=SD√1–ICC. Furthermore, the following equation was used to calculate MDC: MDC=SEM×Z×√2. MDC can be calculated as a percentage to determine the actual relative changes after treatment or between frequent measurements during the time. MDC%=(MDC÷mean) ×100, which mean is the mean score of all repeated measurements. ^22^ If MDC% is less than 30%, it is acceptable and MDC% less than 10 is excellent.^22-24^

***Scoring***

Each item was rated on a 5-point Likert scale (never, rarely, sometimes, often, and always). Scores were converted to a score range of 0-100 using the linear conversion method and the following equation, with a higher score indicating a higher probability of getting involved in RTCs.

$$ Linear Transformation= \frac{Actual Raw score- lowest possible score} {Maximum possible raw score- lowest possible raw score}*100 $$

***The relationship between driver characteristics and the composite scale***

Finally, independent t-test and ANOVA were used to examine the statistical differences between the composite scale and its dimensions with the demographic characteristics of drivers and crash. SPSS-25 and Lisrel 8.8 were used for data analysis.

## Results

***Item generation***

During interviews with 25 experts and 50 car drivers, four main categories were extracted as SCFs influencing the occurrence of RTCs in drivers. Finally, an item pool with 89 items was created (initial version of the instrument). (Appendix 2) 

**Appendix 2:** Instrument with 89 Items to enter the psychometric steps.

Number	Items	always	often	sometimes	rarely	never
Main category: Sociodemographic factors						
Subcategory: Sociodemographic characteristics						
**1**	Life skills and experiences such as time or anger management skills have affected my driving.					
**2**	I am satisfied with my social activities.					
**3**	I go to bed late at night and wake up late in the morning.					
**4**	I drive despite the my car insurance expired.					
**5**	I don't pay my car tax.					
**Subcategory: Social influence**						
**6**	My family's appreciation and respect for traffic laws has affected my driving behavior and performance.					
**7**	I follow the norms of the society and the traffic laws for fear of facing public protest and condemnation of the society.					
**8**	While driving, I am influenced by others (colleagues, friends, passengers, family, other drivers) and drive unsafe.					
**9**	Reducing external pressure, such as the presence of the police on the road, leads me to violate traffic laws. For example, I drive fast on roads where there is no police					
**10**	I find the news media disappointing, and I consider them a factor in creating depression and increasing the number of traffic crashes.					
**11**	Traffic safety training by the community affects my driving.					
**12**	Driving training in schools is inappropriate and non-standard. Because driving schools seek their own profit and issue licenses regardless of driving skills.					
**13**	I am aware of traffic laws and their changes.					
**Subcategory: Beliefs and norms**						
**14**	I respect the customs and norms of society and try not to behave unconventionally while driving.					
**15**	I go to the mosque, pray and fast.					
**16**	I believe that accidents are inevitable.					
**17**	I accept my fate in life.					
**18**	The law blames me for the accident, while I do not blame myself in terms of conscience.					
**19**	If there is no fine in driving, I will violate it and I will not accept it as a crime.					
**20**	I believe that in accidents between cars and other road users, the officer only blames the driver because expects that only the driver should be disciplined and follow the laws.					
**21**	I consider activities such as escaping from the police, speeding to arrive faster or competing to pick up a passenger as a sign of my intelligence and I am proud of it.					
**22**	While driving, especially in the square, I let other drivers cross willingly.					
**23**	I change my driving style when I sit behind the wheel or drive in different environments with different norms.					
**Main category: Personality traits**						
**Subcategory: Sensation seeking and risky behaviors**						
**24**	Out of curiosity, I do dangerous activities while driving.					
**25**	Because of the immediate excitement and the pleasure it brings, I interact with other drivers and do dangerous activities.					
**26**	I am a risk-taker and I believe in "come what may" so, I do anything without considering it right or wrong.					
**27**	I like to be a socially strong person and show my physical abilities by doing strange movements while driving, even if they are dangerous.					
**28**	I like to compete and do whatever it takes to win.					
**29**	I like to drive just for fun and entertainment and have a trip without a specific schedule, route and time.					
**30**	I do not see breaking the law and reckless driving as a sign of bad driving.					
**Subcategory: Violence and anger**						
**31**	I express my protest against life and work problems or existing conditions by engaging in aggressive driving behaviors.					
**32**	I do not respect the right of way in driving. That's why I sometimes get into verbal conflicts with others.					
**33**	Violations of other road users, such as crossing a red light, make me angry, and affect my driving behavior and performance.					
**34**	I frequently blow horns and lights for other drivers to keep them out of my driving route.					
**35**	I get angry when someone makes an ugly behavior towards my driving, such as honking for no reason.					
**Subcategory: Anxiety and lack of time management skill**						
**36**	I'm an anxious person and I get stressed when I'm stuck in a traffic jam.					
**37**	I have been isolated due to the conditions caused by Covid 19 disease. For this reason, the ground for stress in me is ready.					
**38**	I do not have self-confidence. I feel that if I have to deal with another driver, I will lose my spirit and not be able to overcome the problems.					
**39**	I act immediately and without thinking in a moment.					
**40**	My tolerance threshold decreases and I get angry quickly with the behavior of others.					
**Subcategory: Selfishness and individualism**						
**41**	I believe that I am unique in driving. So I just think about myself and the fate of others does not matter to me.					
**42**	I feel like the owner of road and I think I am the king of the road.					
**43**	Because of the sense of power behind the wheel, I make strange moves, interact with other drivers, and consider their activities stupid or inappropriate.					
**44**	I do not trust anyone.					
**45**	I have too much confidence in my driving skills.					
**Subcategory: Locus of control**						
**46**	I consider God's will as the cause of the accident.					
**47**	I consider the cause of the accident to be lack of driving skills, carelessness or error of the other drivers.					
**48**	I think the person I had a crashed with is an opportunistic, tries to blame me by staging and forging documents in order to use the benefits of insurance.					
**49**	I know the cause of the accident is the inattention of the pedestrian while crossing the street.					
**50**	I consider the cause of the accident to be scrapped, unsafe and nonstandard cars.					
**51**	I think the cause of the accident is the unsafe roads.					
**52**	I blame bad weather (such as slippery road on a rainy day) or strong sunlight for my car accident.					
**Subcategory: Risk Perception and hazard monitoring**						
**53**	I am often afraid of new or unexpected situations, and I refuse to do something if I think it is illegal.					
**54**	I often waive my rights to avoid arguing.					
**55**	As a wise person, I avoid risky activities and have no interest in experiencing new and exciting emotions.					
**56**	Before going on a trip, I make sure to plan my trip and check everything in the car.					
**57**	I think carefully about various issues, I examine all the pros and cons before decision making, and plan my work regularly and carefully.					
**58**	I try hard to always be on the lookout for dangers, even when it is not very necessary.					
**59**	I sacrifice today's joy for future success and I work hard.					
**Main category: Driver behavior**						
**Subcategory: Inadequate adherence to traffic laws**						
**60**	I do not wear a seat belt.					
**61**	I use my mobile phone while driving.					
**62**	I eat and drink while driving.					
**63**	I do not pay attention to the traffic lights. For example, I intentionally pass a red light.					
**64**	For various reasons, such as wanting to speed, compensating for delays, achieving the target income sooner, lack of speed camera and police, making others angry or venting anger, I do not follow the legal speed limit.					
**65**	I do not keep a safe distance from the cars around, and move very close to them.					
**66**	Without flasher, I suddenly change direction while driving and turn left or right.					
**67**	I drive long hours non-stop with fatigue and drowsiness.					
**68**	I overtake illegally. For example, I overtake vehicles moving within the speed limit, I overtake on a hill or blind spot, I overtake on the right.					
**69**	I do not respect others while driving. I have no forgiveness and I do not respect the right of way. For example, I drive at an intersection in such a way that the driver who has the right of way is forced to stop and allow me to pass.					
**70**	I do not pay attention to driving signs. For example, on a one-way street, I intentionally drive in the opposite direction, ignore stop signs, park and turn left or right in a restricted area.					
**71**	To escape the traffic jam, I drive in a zig zag manner or drive on the road shoulder.					
**72**	I reverse gear in unauthorized places, such as on a bridge.					
**73**	To disembark the passenger, I open the car door from the left in the middle of the street.					
**Subcategory: Insist on violation**						
**74**	I insist on violating due to lack of patience, and haste to reach the desired destination or income sooner					
**75**	Life and work Problems lead to my mental distress, so I try to circumvent the laws and commit violations to get rid of these pressures.					
**76**	In some cases, there is a conflict between my accepted moral principles and traffic laws and regulations. Therefore, because I adhere to my moral principles and do not trust the laws, I commit the offense.					
**77**	The ugliness of transgression is gone for me and I do not believe in obeying the laws, so I do not deliberately obey them.					
**Subcategory: Resentment and revenge**						
**78**	I do not feel secure in my job. Therefore, I mistreat the passenger to take revenge.					
**79**	I am disappointed in the government, I consider the government's policies and actions towards drivers inappropriate. So I take revenge and break the traffic laws and regulations.					
**80**	I will compensate for the injustices and lies in the society, my insults, humiliations, failures and failures by performing risky behaviors and breaking the traffic laws and regulations.					
**Subcategory: Mental preoccupation and distraction**						
**81**	I get distracted and my driving accuracy decreases due to a variety of activities such as using mobile phones, listening to music, changing the radio wave, searching to find a passenger, getting fare from the passenger, the presence of a child in front of the car, colorful advertisements, looking outside or in the mirror,.					
**82**	Economic problems and lack of public welfare, family and work problems affect my mind and make it impossible for me to be accuracy and focused enough while driving.					
**83**	While pushing the car backwards or in reverse gear, I do not see a passerby behind my car and I collide with her/him.					
**84**	I neglect to service my car on time.					
**Main category: Driver performance**						
**Subclass: Perceptual-motor skills**						
**85**	While driving, I always try to put myself in the place of other drivers so that I can predict their behavior and performance.					
**86**	I predict the traffic situation ahead and drive with mastery.					
**87**	I have enough competence and skills to drive in critical situations and different weather conditions and I can react appropriately at the right time.					
**Subcategory: Safety Skills**						
**88**	When driving, I pay attention to other road users and give priority to their safety and comfort.					
**89**	Issues related to safety and observe the laws and regulations are institutionalized in me and I always tend to behave safely.					

***Face and Content Validity***

In qualitative face validity, 14 items were corrected, while in quantitative face validity, one item was removed due to the impact score <1.5; thus, 88 items were included in the content validity. In the qualitative content validity, according to the experts' opinion, five items were omitted, and four items examining two different topics conceptually were written as separate items. Thus, at the end of this step, the number of items was reduced to 87. In the quantitative content validity, according to the Lawshe table (the evaluation of 10 experts), 30 items were omitted due to CVR <0.62. In the CVI, after calculating I-CVI and Kappa coefficient (K), nine items were removed due to obtaining an index <0.79; and the number of items reached 48. S-CVIAve was 0.92. 

***Item analysis***

At this step, Cronbach's alpha was 0.873. In the inter-item correlation matrix, two items were removed due to a correlation >0.8; and 15 items were deleted due to a correlation ≤2 based on the corrected item-total correlation. Finally, the instrument entered the construct validity with 31 items.

***Construct Validity***

**Principal Component Analysis (PCA)**

**Figure 1:**
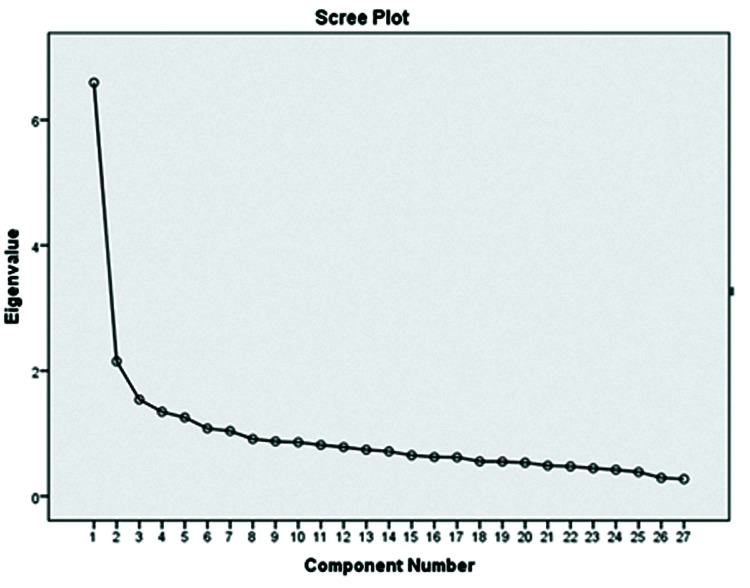
Scree Plot

The 31 items were subjected to using PCA with varimax rotation. The most interpretable solution factors were created using 7 factors, as shown by eigenvalues and scree plot (Figure 1). KMO with a value of 0.872 showed good sampling adequacy, and Bartlett's test of sphericity was significant at p<0.0001 (x^2^ = 3709.540, p<0.0001). 

At this step, the items with a high correlation with each other were placed inside a factor, and four items having a factor load < 0.4 were deleted. The analysis revealed 7 factors that explained 55.56% of the total variance. The factor loadings are shown in Table 1. 

**Table 1:** Factor loadings of the 27 items in the sociocultural scale.

Factors Items	Inadequate adherence to traffic laws	Risk-taking	Beliefs and norms	Driver performance	Irritability and anger	Adherence to traffic laws	External locus of control
To disembark the passenger, I open the car door from the left in the middle of the street.	0.68						
I reverse gear in unauthorized places, such as on a bridge.	0.64						
Without flasher, I suddenly change direction while driving and turn left or right.	0.61						
I do not keep a safe distance from the cars around, and move very close to them.	0.55						
Life and work problems lead to my mental distress, so I try to circumvent the laws and commit violations to get rid of these pressures.	0.52						
I use my mobile phone while driving.		0.79					
I eat and drink while driving.		0.66					
I also follow the laws where there is no police.		-0.63					
I drive long hours non-stop with fatigue and drowsiness.		0.54					
I am careful to plan before traveling, and check all the parts of the car.		-0.45					
I consider activities such as escaping from the police, speeding to arrive faster or competing to pick up a passenger as a sign of my intelligence and I am proud of it.			0.61				
It is interesting for me to do dangerous activities while driving.			0.59				
I respect the customs and norms of society and try not to behave unconventionally while driving.			-0.55				
If I think it's illegal to do something, I refuse to do it.			-0.50				
To escape the traffic jam, I drive in a zig zag manner or drive on the road shoulder.			0.49				
I make dangerous movements while driving due to being in a hurry.			0.47				
I have enough competence and skills to drive in a critical situations and I can react appropriately at the right time.				0.79			
I predict the traffic situation ahead and drive with mastery.				0.75			
When driving, I pay attention to other road users and give priority to their safety and comfort.				0.65			
I get angry when someone makes an ugly behavior towards my driving, such as honking for no reason.					0.74		
I get angry that other road users do not follow the traffic laws.					0.67		
I get stressed when I get stuck in traffic.					0.58		
I frequently blow horns and lights for other drivers to keep them out of my driving route.					0.53		
I pay attention to traffic signs.						0.82	
I respect the right of way in driving.						0.80	
I blame bad weather (such as slippery road on a rainy day) or strong sunlight for my car accident.							0.77
In a pedestrian accident, I blame his/her for the accident.							0.61

Factor 1, named “Inadequate adherence to traffic laws”, explained 9.59% of the variance and consisted of 5 items. Factor 2, named “risk-taking”, explained 9.47% of the variance and consisted of 5 items. Factor 3, named “Beliefs and norms”, explained 9.10% of the variance and consisted of 6 items. Factor 4, named “Driver performance”, explained 7.79% of the variance and consisted of 3 items. Factor 5, named “irritability and anger”, explained 7.42% of the variance and consisted of 4 items. Factor 6, named “adherence to traffic laws”, explained 6.71% of the variance and consisted of 2 items. Factor 7, named “External locus of control”, explained 5.45% of the variance and consisted of 2 items.

**Confirmatory factor analysis**

CFA was performed using maximum likelihood estimation procedures to assess the fitness of the sociocultural scale. The model fit of the factorial structure obtained through PCA was examined. The results of Table 2 shows that the indices of the model fit are satisfactory.

**Table 2:** Goodness-of fit indices for model of the sociocultural scale.

Fit indices	Result	Acceptable range
Chi-squared P-value ( χ 2 P-value)	0.0	>0.05
Root Mean Square Error of Approximation (RMSEA)	0.052	Good <0.08
		Average <0.08-0.1
		Weak > 0.1
Minimum Discrepancy Function by Degrees of Freedom divided (CMIN/DF)	2.32	Good <3
		Acceptable <
Normed Fit Index (NFI)	0.92	> 0/9
Non-Normed Fit Index (NNFI)	0.95	> 0/9
Parsimony Normed Fit Index (PNFI)	0.80	> 0/5
Comparative Fit Index (CFI)	0.96	> 0/9
Incremental Fit Index (IFI)	0.96	> 0/9
Relative Fit Index (RFI)	0.91	> 0/9
Standardized Root Mean Square Residual (SRMR)	0.054	< 0/1
Goodness of Fit Index (GFI)	0.91	> 0/9
Adjusted Goodness of Fit Index (AGFI)	0.88	> 0/8

**Discriminant analysis**

The results of Table 3 show that there is a significant difference between the two groups of accident-involved drivers and accident-free drivers in terms of scores obtained in the composite scale and its dimensions.

**Table 3:** Discriminant validity of scale for two groups of accident-involved drivers and accident-free drivers.

Dimensions	Accident-involved drivers		Accident-free drivers		Pvalue
	Mean	SD	Mean	SD	
Inadequate adherence to traffic laws	36.27	20.60	0.16	0.86	< 0.001
Risk-taking	48.71	17.24	1.38	2.97	< 0.001
Beliefs and Norms	36.94	23.62	1.87	4.92	< 0.001
Driver performance	38.79	26.91	1.59	5.57	< 0.001
Irritability and Anger	57.68	17.39	7.44	8.66	< 0.001
Adherence to traffic laws	45.42	26.76	0.42	2.28	< 0.001
External locus of control	48	28.87	10	18.24	< 0.001
Scale	43.49	11.95	2.45	1.36	< 0.001

***Reliability***

Statistical results of Cronbach's alpha, Theta, Omega coefficients and ICC for determining reliability of the sociocultural scale are shown in Table 4 . The reliability of the entire questionnaire for the above-mentioned indices were 0.82, 0.96, 3.07, and 0.80, respectively; which indicated good reliability of the instrument. 

**Table 4:** The Cronbach alpha, theta, omega coefficients, intra-class correlation coefficient, and absolute reliability of sociocultural scale for car drivers.

Dimensions	Number of items	Cronbach's alpha	θ	Ω	ICC	CI= 95%	SEM	MDC	MDC%
Inadequate adherence to traffic laws	5	0.69	1.06	1.27	0.74	0.45-0.87	1.18	3.27	47
Risk-taking	5	0.74	0.66	1.30	0.80	0.58-0.90	0.92	2.55	17
Beliefs and Norms	6	0.67	0.41	1.32	0.80	0.57-0.90	0.91	2.52	17
Driver performance	3	0.72	0.38	1.18	0.90	0.80-0.95	0.56	1.55	11
Irritability and Anger	4	0.61	0.26	1.22	0.71	0.40-0.86	1.66	4.60	47
Adherence to traffic laws	2	0.80	0.14	1.11	0.85	0.70-0.93	0.46	1.27	13
External locus of control	2	0.49	0.07	1.10	0.79	0.56-0.90	0.74	2.05	42
Scale	27	0.82	0.96	3.07	0.80	0.59-0.90	3.27	9.06	12

ICC: Intra-class Correlation Coefficient CI: Confidence Interval SEM: Standard Error of Measurement MDC: Minimal Detectable Change

***Absolute reliability***

Absolute reliability was calculated by SEM, and MDC was estimated (Table 4 ).

***The Relationship between driver characteristics and the composite scale***

Table 5 shows the relationship between driver characteristics with the composite scale and its dimensions. There was a significant difference between the scores of composite scale in drivers' characteristics such as age, marital status, having children, years of driving experience, vehicle ownership, type of vehicle, alcohol and cigarette consumption, and drug addiction. Thus, the composite scale score in drivers under 40 years, single or married without children, with no sufficient driving experience, without vehicle ownership especially in vehicles manufactured by Iran Khodro Company, with a history of smoking, alcohol and drug abuse was significantly higher than those in their other subgroups. 

**Table 5:** Association between drivers’ characteristics and sociocultural scale.

Characteristics		F1	F2	F3	F4	F5	F6	F7	Sociocultural scale	p-value scale
					Mean Score					
Age (years)	40>	13.80	26.11	15.59	15	38.98	13.04	27.71	20.93	0.000
	40≤	8.91	16.65	8.44	9.59	35.19	9.59	21.48	14.80	
Gender	Male	10.64	19.97	11.07	10.68	35.39	10.51	22.60	16.66	0.062
	Female	10.13	21.45	11.59	18.69	43.01	12.31	32.50	19.54	
Marital status	Single	13.60	25.14	16.14	12.66	38.81	11.21	24.55	20.22	0.016
	Married	10.11	19.38	10.35	11.59	36.02	10.67	23.80	16.53	
Child	Yes	9.55	18.94	9.48	10.35	35.60	9.42	22.20	15.77	0.000
	No	14.07	24.32	16.75	16.42	39.09	15.24	29.69	21.32	
Place of residence	City	10.31	20.22	11.06	11.54	36.70	11.25	23.68	17.02	0.96
	Suburbs	11.30	16.80	13.62	10.62	35.45	6.25	30.88	16.77	
	Rural	14.23	23.70	9.24	16.43	32.41	8.17	18.65	17.60	
Level of education	illiterate	0.000	1.87	28.57	18.18	3.57	0.000	32.50	10.50	0.097
	primary school	13.62	16.25	12.19	10.65	45.44	14.22	18.27	18.25	
	Middle and high school	9.22	19.18	11.98	10.11	34.23	7.33	18.46	15.70	
	Diploma	10.04	19.62	10.11	10.39	35.27	9.66	24.87	16.25	
	College education	11.74	23.28	12.04	16.02	37.71	14.85	27.25	19.24	
Certificate type	Basic certificate one	12.21	22.25	10.94	9.63	36.76	11.68	19.02	17.40	0.82
	Basic certificate two	10.42	19.96	11.16	11.95	36.36	10.65	24.40	17	
Driving experience (Years)	10≥	15.14	26.45	18.01	16.74	42.79	14.41	31.26	22.80	0.000
	11-20	10.45	21.71	10.86	12.26	35.50	11.15	23.84	17.22	
	20<	8.23	15.30	7.50	8.19	34.36	8.55	19.67	13.74	
Vehicle ownership	Own vehicle	10.19	19.72	10.20	11.11	35.81	9.92	23.24	16.42	0.001
	Others’ vehicle	13.47	23.53	18.15	16.44	40.76	16.94	28.89	21.66	
Type of vehicle	SAIPA Company	8.66	17.39	9.50	9.62	36.80	8.42	21.45	15.25	0.045
	Iran Khodro Company	11.11	20.83	11.73	12.03	35.18	12.04	24.79	17.42	
Income (million Rials)	30>	10.19	15.94	10.09	13.18	37.69	9.43	21.09	15.98	0.488
	30≤	9.88	21.16	10.78	10.53	36.16	10.49	24.93	16.88	
Smoking	Yes	14.73	24.66	14.26	12.06	39.02	11.88	24.54	19/99	0.000
	No	8.94	18.39	9.90	11.61	35.36	10.30	23.65	15/87	
Alcohol consumption	Yes	30.27	37.98	28.83	22.43	47.67	31.52	38.91	33.61	0.000
	No	9.63	19.31	10.29	11.22	35.85	9.74	23.18	16.24	
Addict	Yes	18.67	34.84	20.23	15.34	45.64	25	28.75	26.65	0.001
	No	10.31	19.68	10.84	11.62	36.09	10.27	23.75	16.72	
Fine	Yes	10.53	20.96	10.99	10.54	36.76	11.29	23.56	17.10	0.860
	No	10.71	18.29	11.51	14.58	35.53	9.45	24.72	16.89	

Statistical significant = p < 0.05

**Crash involvement by sociocultural subscales**

The results of Table 6 show that the scores obtained from the composite scale and each of its seven subscales are higher among accident-involved drivers than those in accident-free drivers. There was a statistically significant difference between the scores of the subscales of inadequate adherence to traffic laws, risk-taking, beliefs and norms, driver performance, adherence to traffic laws, as well as composite scale in the two groups.

**Table 6:** Association between sociocultural scale and their subscale with crash involvement for the influence of structural characteristics.

Dimensions	Crash Involvement				P
	No		Yes		
	mean	SD	mean	SD	
Inadequate adherence to traffic laws	13.38	16.74	9.24	13.55	**0.003**
Risk-taking	23.60	19.34	18.52	16.39	**0.002**
Beliefs and Norms	14.21	18.15	9.67	13.04	**0.001**
Driver performance	14.35	20.54	10.48	16.08	**0.022**
Irritability and Anger	38.81	22.13	35.34	29.57	**0.077**
Adherence to traffic laws	14.96	22.67	8.72	18.95	**0.001**
External locus of control	25.92	27.47	22.94	23.46	**0.209**
Scale	20.02	14.49	15.61	9.84	**0.000**

## Discussion

The aim of the present study was to develop a valid and reliable instrument for SCFs to identify risky drivers and predict RTCs in Iranian drivers. The initial questionnaire was developed based on data from a qualitative study and extensive investigation of the existing literature on SCFs affecting the RTCs in the drivers. Factor analysis identified seven factors including: inadequate adherence to traffic laws, risk-taking, beliefs and norms, driver performance, irritability and anger, adherence to traffic laws, and external locus of control, which seem to be a good extension of SCFs of today's drivers. Psychometric properties and usability of the instrument were examined, and its validity and reliability were proven.

Inadequate adherence to traffic laws, which includes non-compliance with traffic laws, has been extracted as one of the most important SCFs affecting the occurrence of RTCs. Inadequate adherence to traffic rules includes several types of dangerous behaviors that drivers exhibit while driving, such as stopping and reversing gear in unauthorized area and tailgating. This subscale is similar to past extracted factors in the driving violations’ dimension for predicting self-reported accidents in the aggressive driving scale. ^25^ Past studies have indicated that these violations are mostly committed for retaliatory actions and associated with the occurrence of RTCs.^26-28^

In the present study, risk-taking factor along with high-risk activities such as eating, drinking and using cell phones while driving, as well as driving during fatigue was extracted as one of the effective SCFs in the occurrence of RTCs. These activities may lead to distracted driving. Researchers have found that the drivers who become distracted while driving underestimate the potentially dangerous consequences of their distraction.^29^ This finding is consistent with a large number of empirical studies, in which risk-taking and attitudes toward high-risk driving positively predict risky driving behaviors and RTCs.^30-32^ Other items that appear in this factor are having a goal and traffic discipline, which have a negative relationship with the occurrence of RTCs. Past studies have suggested that driving without planning and traffic discipline, and decision making without considering all the positive and negative consequences, predispose the situation to RTCs.^33,34^

Beliefs and norms emerged as a separate factor among SCFs influencing the occurrence of accidents. People who believe in values and moral principles are less likely to engage in deviant behaviors. In the research of Ansari et al., belief in compliance with traffic laws was identified as a predictor of RTCs. Also, according to the social control theory, acceptance and participation in social activities, respect for moral values and cultural norms of society demonstrate a sense of belonging and responsibility to society. People who are more committed to society appear not to deviate due to fear of losing their achievements.^35^ In other words, human behavior is largely limited by laws governing specific situations and social interactions. However, people do not always abide by the law, and this seems to reduce the safety provided by the law, and increases the likelihood of RTCs.^36^

Driver performance or driving skills is another influential factor in RTCs. Similar to previous researches, the present study clearly showed safety and perceptual-motor skills as two important factors of driving skills. With these skills, drivers can perform risk assessment, information processing and vehicle control. Perceptual-motor skills and safety skills are positively and negatively related to RTCs, respectively.^37-38^ Similarly, Xu et al. found that driver performance, as an important factor, has an impact on driving behavior and consequently on RTCs.^39^ Drivers can reduce traffic hazards by balancing safety and perceptual-motor skills and creating cautious situations and thus predict potential road hazards.^40^

Another influential factor in RTCs is the driver's irritability and anger, which is one of drivers' mood behaviors, and can be affected by emotions. Irritated and angry drivers cannot control their emotions in the face of external stimuli such as repeated honking and flashing of other drivers, lack of respect for traffic laws by other drivers or even when stuck in a traffic jam; thus, they may make decision that puts them or others at risk for RTCs.^3,41^ This result confirms the previous findings^27,42-44^ in which the driver's mood and anger are recognized as an individual factor in the occurrence of RTCs. Moreover, these behaviors can negatively impact on driver performance that predicts dangerous consequences and accidents, which is consistent with other studies.^45^

In line with previous reports, adherence of drivers to traffic laws has been introduced as one of the effective factors in preventing RTCs^27,46,47^ Since inadequate adherence to traffic laws has been described as the first known effective factor in the occurrence of RTCs in this study, the factor of adherence to traffic laws was extracted as a separate factor. It seems that it has the opposite effect on unsafe and risky driving behaviors and prevents many dangerous situations and accidents.

The last influential factor is the external locus of control. In the field of traffic psychology, locus of control has an important role in determining risky driving behaviors and accidents.^48^ According to preliminary studies, people with an external locus of control are the most dangerous individuals in the road safety, because they do not take sufficient safety and precautionary measures to prevent RTCs.^49^ One of the external loci of control is environmental factor^48^ such as bad weather accounted for the drivers’ high-risk behaviors and accidents.^50^ Interestingly, Sun et al. found that attributing the cause of accidents to the environmental factors increases drivers' tendency to adopt an angry driving style.^48^ This may be explained by the fact that drivers who engage in angry driving are provoked in bad weather or road condition.^35^ Drivers sometimes blame other road users for their accidents. In most previous studies, other drivers were introduced as an effective external locus of control in causing accidents.^48,50^ However, in the phase 2 of the present study and during interviews with drivers, pedestrians were recognized as one of the most important factors in the external locus of control. Although the drivers were at fault for the accident with a pedestrian in most situations, they did not believe much in this matter. 

The present study was the first research to identify SCFs predicting RTCs in car drivers in the form of designing an instrument in Iran, which has used strong methodological and statistical approaches to provide a valid instrument. Based on the results, this instrument can be useful to distinguish between accident-involved drivers and accident-free drivers. Moreover, it may be beneficial for developing interventions for drivers who are prone to RTCs in Iran.

**Limitations and strengths of the study**

There are limitations in the present study. First, convenience sampling was used to select driver participants; therefore, the results may not be generalizable to the entire population of Iranian drivers. Thus, larger and more representative samples should be included in future studies. Second, since the questionnaires were filled out self-reportedly by drivers, the correctness of the answers may be affected by social convenience. The risk of this bias was partially alleviated by filling out a questionnaire in absence of the researcher. However, several studies have suggested that driver's self-assessment was valid and widely used. ^11,51^ Third, the instrument was used to predict self-reported accidents, not official crash records; hence, there may be a difference between the two data sources. It is recommended to use a combination of both data sources for future studies. One of the most reliable data sources for collecting crash records is the police. Since in many minor accidents, without the presence of the police, the parties to the accident resolve the issue, it may underestimate the number of accidents. Fourth, the naming of factors was based on the choice of researchers. Lack of similar studies in the field of SCFs affecting the occurrence of RTCs has led to some problems in the classification and naming of factors; therefore, it is recommended that more detailed studies with different statistical populations be performed by other researchers to achieve more reliable results.

Despite the mentioned limitations, there was a lack of instrument to examine the SCFs predicting RTCs in drivers; thus, the current instrument helped to promote knowledge in this field, and can be a reference for developing more effective instrument in the future. Another strength was extensive field-sampling, because researcher tried to increase the accuracy of collected data by actively participating in the gathering of drivers and explaining the objectives of the study individually for each of them; and employ different drivers in terms of gender, age, driving experience and level of education to minimize the sampling bias. 

## Conclusion

In conclusion, the validity and reliability of the designed instrument to measure SCFs predicting RTCs were satisfactory. The present study was the first research that examined the impact of SCFs on the incidence of RTCs in the Iranian drivers. This scale seems to be very useful instrument for assessing and screening out accident-prone drivers. Composite scale had good reliability, but some of its subscales needed further reliability due to their low internal consistency. Correlation results indicated that composite scale and most of its subscales can predict RTCs. However, self-report scales alone cannot identify all individuals who have reported an accident.^52^ Therefore, it is necessary to use a combination of self-report and official data sources in future research.

**Acknowledgments**

We acknowledge all the participants who helped us in this study.
